# Trends in Industry Payments to Diabetologists and Endocrinologists in the United States During the COVID-19 Pandemic

**DOI:** 10.7759/cureus.32643

**Published:** 2022-12-17

**Authors:** Anju Murayama, Sae Kamamoto, Hiroaki Saito, Tetsuya Tanimoto, Akihiko Ozaki

**Affiliations:** 1 School of Medicine, Tohoku University, Sendai, JPN; 2 School of Medicine, Hamamatsu University School of Medicine, Hamamatsu, JPN; 3 Gastroenterology, Soma Central Hospital, Soma, JPN; 4 Internal Medicine, Navitas Clinic, Tokyo, JPN; 5 Surgery, Teikyo University, Graduate School of Public Health, Tokyo, JPN

**Keywords:** health policy and economics, public health policy, health public, endocrinologist and diabetologist, covid-19 pandemic, sars-cov-2, covid-19

## Abstract

Introduction

Limited evidence suggests there were substantial amounts of payments from the healthcare industry to diabetologists and endocrinologists in the United States before the coronavirus disease 2019 (COVID-19) pandemic period. However, there is no study on how these industry payments changed during the COVID-19 pandemic. This study aimed to evaluate trends in non-research industry payments to physicians specializing in diabetology and endocrinology in the United States during the COVID-19 pandemic.

Methods

Using the open payments database between 2013 and 2021, we examined trends in general payments made to physicians whose primary specialty was diabetology and endocrinology by the healthcare industry in the United States. Trends in industry payments during the COVID-19 pandemic were evaluated by interrupted time series analysis with generalized estimating equation models.

Results

Of 7965 active endocrinologists and diabetologists, 6991 (87.8%) received one or more general payments from the healthcare industry in the United States between August 2013 and December 2021. Median per-physician payments were $116.68 (interquartile range (IQR): $41.66-$390.00) before the COVID-19 pandemic period and $97.91 (IQR: $32.81-$314.04) during the COVID-19 pandemic period. Monthly per-physician payments, the number of per-physician payments, and the number of physicians receiving payments decreased by 61.0% (95% confidence interval (95% CI): 58.1%-63.7%, p<0.001), 59.2% (95% CI: 57.9%-60.4%, p<0.001), and 39.7% (95% CI: 38.3%-41.0%, p<0.001) at the onset of the COVID-19 pandemic (March 2020), compared to those before pandemic period, respectively.

Conclusion

The non-research payments to endocrinologists and diabetologists from the healthcare industry sharply decreased by about 60% in payment amounts due to the COVID-19 pandemic in the United States.

## Introduction

In response to increased public demand for greater transparency in physician-industry financial relationships, the Physician Payment Sunshine Act was enacted in the United States in 2010 and the accompanying open payments database was incepted in 2013. The Sunshine Act mandates pharmaceutical and medical device manufacturers to disclose nearly all financial transfers made to physicians on the federal online database, namely, the open payments database [1]. Many previous analyses showed that payments from the healthcare industry influenced physicians’ prescribing patterns [2,3].

Before the coronavirus disease 2019 (COVID-19) pandemic, there were considerable financial interactions between endocrinologists and diabetologists and the healthcare industry in the United States. One study reported that endocrinologists and diabetologists accepted 3.5 times larger non-research payments such as meals, travel fees, lecturing compensations, and consulting fees from the healthcare industry compared to those to internal medicine physicians between 2015 and 2017 [4]. These non-research payments were positively associated with the increase of sponsored drugs in the fields of endocrinology and diabetology [5,6]. The sudden COVID-19 pandemic substantially altered lifestyles, economic activities, and healthcare systems. The physician-industry relationships have also been restricted due to the COVID-19 pandemic. Although the interruption in industry payments to endocrinologists and diabetologists was expected during the COVID-19 pandemic, little is known about the trends in endocrinologist and diabetologist-industry financial relationships during the pandemic so far.

## Materials and methods

Study design, study participants, and data collection

This retrospective population-based cohort study aimed to evaluate the trends in general payments made to endocrinologists and diabetologists from the healthcare industry during the COVID-19 pandemic. This study included all active physicians whose primary specialty was classified as “Endocrinology, Diabetes and Metabolism” in the National Plan and Provider Enumeration System (NPPES). Physicians who were deactivated and newly activated between August 2013 and December 2021 were excluded from the study population. We matched the NPPES database and the open payments database by the National Provider Identifier number. All general payments made to endocrinologists and diabetologists were extracted from the open payments database.

Data analyses

Trends in general payments to endocrinologists and diabetologists during the COVID-19 pandemic were examined monthly by an interrupted time series (ITS) analysis with generalized estimating equation (GEE) models of monthly panel data of general payments at the physician level [7]. The per-physician general payments overall and by payment categories were analyzed by a negative binomial regression GEE model, and the number of physicians receiving payments was analyzed by a linear log-linked GEE with a Poisson distribution, as the payments were right-skewed. The seasonality of monthly payments was adjusted by including the monthly variable. The study period was divided into before (August 2013 to February 2020) and during the COVID-19 pandemic (March 2020 to December 2021) in the monthly ITS analysis, as the national emergency concerning the COVID-⁠19 pandemic was declared in the United States on March 13, 2020 [7,8]. The payments for ownership interests, royalties and licenses, and several newly-added categories in 2021 were excluded, as these payments were substantially distributed to a very small number of physicians.

Ethical clearance

This study was approved by the Ethics Committee of the Medical Governance Research Institute before this study began. As this analysis included only public data, informed consent was waived by the Ethics Committee of the Medical Governance Research Institute.

## Results

Of the 7965 active endocrinologists and diabetologists registered in the NPPES, 6991 (87.8%) received one or more general payments from the healthcare industry in the United States between August 2013 and December 2021. During the pre-pandemic period (August 2013 to February 2020), 2790 to 3470 physicians received general payments each month. The median monthly payments per physician (interquartile range (IQR)) ranged from $80.95 ($31.00-$255.10) in December 2013 to $255.10 ($53.33-$561.6) in March 2017.

**Figure 1:**
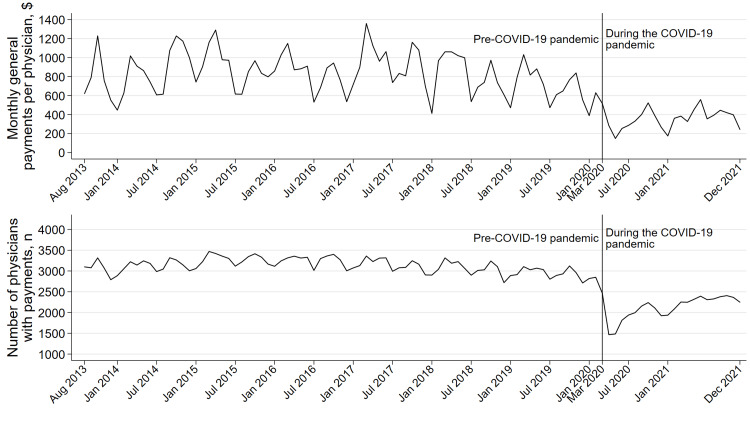
Monthly trend in general payments per physician and number of physicians with payments in the United States between 2013 and 2021. General payments for acquisitions, current or prospective ownership or investment interest, debt forgiveness, long-term medical supply or device loan, and royalty or license were excluded from the monthly ITS analysis, as these payments were outliers and distributed to a very small number of physicians or included since 2021 payment data. The study period was divided into before (August 2013 to February 2020) and during the COVID-19 pandemic (March 2020 to December 2021) in the monthly ITS analysis, as the national emergency concerning the COVID-⁠19 pandemic was declared in the United States on March 13, 2020. ITS: interrupted time series.

In March 2020, 2466 physicians (31.0%) received a median of $72.51 general payments, decreasing from 2849 (35.8%) in the number of physicians receiving payments and $101.8 in median per-physician general payments in February 2020. The median per-physician payments were $116.68 (IQR: $41.66-$390.00) during the pre-COVID-19 pandemic period and $97.91 (IQR: $32.81-$314.04) during the COVID-19 pandemic period. The monthly per-physician general payments, the number of per-physician payments, and the number of physicians receiving payments decreased by 61.0% (95% confidence interval (95% CI): 58.1%-63.7%, p<0.001), 59.2% (95% CI: 57.9%-60.4%, p<0.001), and 39.7% (95% CI: 38.3%-41.0%, p<0.001) at the onset of the COVID-19 pandemic (March 2020), compared to those in the pre-COVID-19 pandemic period, respectively (Figure 1). After the sharp decrease due to the COVID-19 pandemic, the per-physician general payments, the number of per-physician payments, and the number of physicians receiving payments increased by 1.9% (95% CI: 1.5%-2.3%, p<0.001), 2.9% (95% CI: 2.7%-3.1%, p<0.001), and 1.6% (95% CI: 1.4%-1.7%, p<0.001), respectively. However, the industry payments to physicians specializing in diabetology and endocrinology remained at a lower level even in 2021. In 2021, 1923 to 2407 physicians received from $78.28 to $115.19 in median per-physician payments.

During the pre-COVID-19 pandemic period, per-physician general payments, number of per-physician payments, and number of physicians receiving payments slightly decreased by 0.23% (95% CI: 0.13%-0.33%, p<0.001), 0.13% (95% CI: 0.08%-0.18%, p<0.001), and 0.09% (95% CI: 0.06%-0.13%, p<0.001), respectively. During the COVID-19 pandemic, there was no difference in per-physician payments between 2020 and 2021.

**Figure 2:**
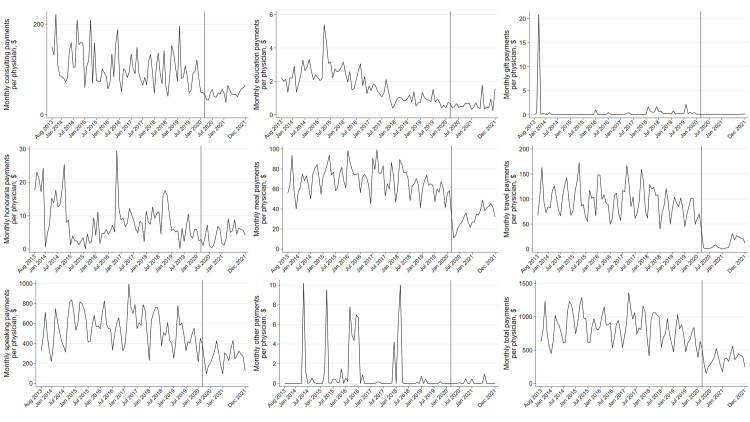
Monthly per-physician payments by payment categories between August 2013 and December 2021. General payments for acquisitions, current or prospective ownership or investment interest, debt forgiveness, long-term medical supply or device loan, and royalty or license were excluded from the monthly ITS analysis, as these payments were outliers and distributed to a very small number of physicians or included since 2021 payment data. The study period was divided into before (August 2013 to February 2020) and during the COVID-19 pandemic (March 2020 to December 2021) in the monthly ITS analysis, as the national emergency concerning the COVID-⁠19 pandemic was declared in the United States on March 13, 2020. ITS: interrupted time series.

**Figure 3:**
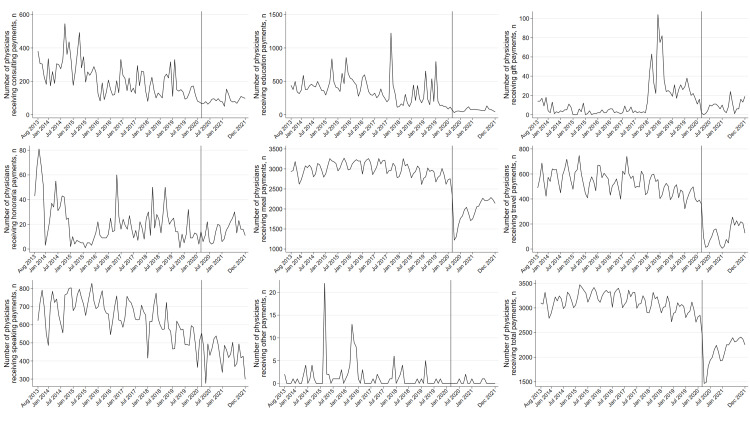
Monthly number of physicians receiving payments by payment categories between August 2013 and December 2021. General payments for acquisitions, current or prospective ownership or investment interest, debt forgiveness, long-term medical supply or device loan, and royalty or license were excluded from the monthly ITS analysis, as these payments were outliers and distributed to a very small number of physicians or included since 2021 payment data. The study period was divided into before (August 2013 to February 2020) and during the COVID-19 pandemic (March 2020 to December 2021) in the monthly ITS analysis, as the national emergency concerning the COVID-⁠19 pandemic was declared in the United States on March 13, 2020. ITS: interrupted time series.

Sensitivity analysis of monthly payments by payment categories revealed that the travel and accommodation payments decreased the most, with a relative change rate of −95.8% (95% CI: −96.5% to −95.0%, p<0.001). The per-physician payment amounts for meals, speaking compensations, consulting, and education decreased by 69.4% (95% CI: 68.2%-70.5%, p<0.001), 55.8% (95% CI: 52.9%-58.4%, p<0.001), 49.7% (95% CI: 28.3%-64.8%, p<0.001), and 38.7% (95% CI: 11.7%-57.4%, p=0.009), respectively (Figure 2). All payment categories other than gift payments had decreased during the pre-pandemic period. The number of physicians receiving payments for gifts, travel, and education decreased by 91.8% (95% CI: 85.6%-95.3%, p<0.001), 80.3% (95% CI: 78.6%-81.8%, p<0.001), and 76.5% (95% CI: 73.9%-78.9%, p<0.001), respectively, though speaking payments only decreased by 15.9% (95% CI: 12.7%-19.1%, p<0.001). Physicians receiving meal payments decreased by 44.8% (95% CI: 43.4%-46.2%, p<0.001) (Figure 3).

## Discussion

This study demonstrated that the non-research payments from the healthcare industry to physicians specializing in diabetology and endocrinology decreased by about 60% in monetary amounts and number of payments and about 40% in the number of physicians receiving payments due to the COVID-19 pandemic in the United States. However, we observed recovering trends in industry payments. Additionally, there were slightly declining trends in industry payments before the COVID-19 pandemic. The payments for travel and accommodation, meals, and speaking compensations decreased in particular at the onset of the COVID-19 pandemic. This study could be the first analysis evaluating trends in industry payments in the fields of diabetology and endocrinology during the COVID-19 pandemic.

The sharp reduction in industry payments was reported in several specialties in the United States [7-9]. We previously reported the decrease in per-physician payments was 52.6% in allergology and 58.6% in infectious diseases due to the COVID-19 pandemic in the United States [7,8]. Inoue et al. found that industry payments decreased by 48.4% in total due to the COVID-19 pandemic [9]. Although Inoue et al. found that payments for consulting and speaking compensations slightly decreased or did not decrease [9], we observed a decrease in both consulting and speaking payments by about half in the field of diabetology and endocrinology, indicating that the endocrinologists and diabetologists with influential positions, namely, key opinion leaders, also could have received fewer payments from the healthcare industry during the COVID-19 pandemic. The reduction in industry payments to endocrinologists and diabetologists raises the question of whether this will lead to a decline in industry influence on physicians’ prescriptions and patient care. Numerous studies have illustrated that industry payments to physicians influence physicians’ therapy selections and increase healthcare costs [2], which sometimes led to harmful and lower-quality therapies for patients [10,11]. Endocrinologists and diabetologists were also influenced by industry payments [5,12]. Future studies must address whether the reduction in industry payments to endocrinologists and diabetologists due to the COVID-19 pandemic could lower industry influence on physicians’ clinical practice.

This study included several limitations. First, we could not rule out the possibility of inaccuracies in the open payments database. As Feng et al. suggested, only a small number of physicians disputed their payment data [13]. Second, the interrupted time series analysis only evaluated the trend in industry payments by the influence of the COVID-19 pandemic. Therefore, there would be other unmeasured confounding factors influencing the trend in industry payments to endocrinologists and diabetologists in the United States. However, our analysis of general payments to all registered endocrinologists and diabetologists enables us to assess the whole trend in industry payments in the fields of diabetology and endocrinology in the United States.

## Conclusions

Despite that this study included several limitations, this first analysis of industry payments to endocrinologists and diabetologists during the COVID-19 pandemic illustrated that the non-research industry payments to endocrinologists and diabetologists sharply decreased by about 60% due to the COVID-19 pandemic. There were recovering trends in the industry payments right after the sharp reduction, but the industry payments remained at a lower level even in 2021. All endocrinologists and diabetologists should pay attention to their clinical practice was influenced by industry payments before the COVID-19 pandemic. Future studies should elucidate the industry influence on endocrinologists' and diabetologists' practice remains during the COVID-19 pandemic.
